# Equipment for Testing Measuring Devices at a Low-Level Radon Activity Concentration

**DOI:** 10.3390/ijerph17061904

**Published:** 2020-03-15

**Authors:** Eliska Fialova, Petr P. S. Otahal, Josef Vosahlik, Monika Mazanova

**Affiliations:** 1 National Institute for Nuclear, Biological and Chemical protection (SUJCHBO, v.v.i.), 262 31 Milin, Czech Republic; otahal@sujchbo.cz (P.P.S.O.); vosahlik@sujchbo.cz (J.V.); 2 Czech Metrological Institute (CMI), 102 00 Prague, Czech Republic; mmazanova@cmi.cz

**Keywords:** radon, radon chamber, radon source, low-level radon activity concentration

## Abstract

The National Institute for Nuclear, Biological, and Chemical Protection, under the European project 16ENV10 MetroRADON (the European metrology program for innovation and research, EMPIR), has developed unique equipment for the testing of measuring devices at low-level radon activity concentrations. The equipment consists particularly of an airtight low-level radon chamber (LLRCH) with an inner volume of 324 liters; a Rn-222 type RF 5 flow-through source with a Ra-226 activity of 4.955 kBq developed by Czech Metrological Institute within the above-mentioned project; and a pressure vessel as a radon-free air source. The mass flow controller of the Bronkhorst EL-Flow type is a part of the apparatus and ensures the requested airflow through the radon source—partialized if necessary—through the chamber. The homogeneity of the atmosphere in the chamber is ensured by means of a continuously regulated fan (airflows in the range of 0.1–3.5 m·s^−1^ can be established). Another important chamber component is the measuring device of climatic conditions, since temperature, air pressure, and relative humidity must be determined. The construction of the equipment allows the time-stable radon activity concentration to be maintained at a precise level for several days. Radon concentration values can be arbitrarily and continuously set in the range from 100 Bq·m^−3^ to 300 Bq·m^−3^.

## 1. Introduction

Radon 222 is a naturally occurring radioactive noble gas as a member of the natural uranium–radium family. The latter is found in variable amounts in rocks and soils. Radon comes through the cracks and spreads into the atmosphere of the dwellings and other indoor places. The amount of radium 226 in the ground and the porosity of bedrock affects the level of indoor radon activity concentration [1].

Non-gaseous, short-lived radon-daughter products attached to aerosols are present in inhaled air. The settling of such aerosols in the respiratory tract leads to the alpha irradiation of the bronchial system [1].

The WHO [2] estimated that between 3% and 14% of all lung cancer cases in the world are caused by radon (or radon decay products, respectively) depending on the average of the radon level in the country. This corresponds to several thousand people dying per year of lung cancer caused by radon exposure in Europe.

Radon measurement techniques to evaluate the radon concentration in dwellings are simple, efficient and precise. The levels of relevant concentrations in European dwellings have been lowered (300 Bq·m^−3^); however, there is still a need to develop and/or improve the accuracy of calibration procedures for existing commercial radon monitoring practices [3,4,5].

The evaluation and calibration of measuring devices for radon and radon daughter products (RnDP) requires stable conditions of radon activity concentration. In the assignment of the Project 16ENV10 MetroRADON, keeping a stable radon activity concentration at a precise level for several days was the main goal of the development of new equipment for testing measuring devices at low-level radon activity concentrations.

## 2. Material and Methods

**Figure 1:**
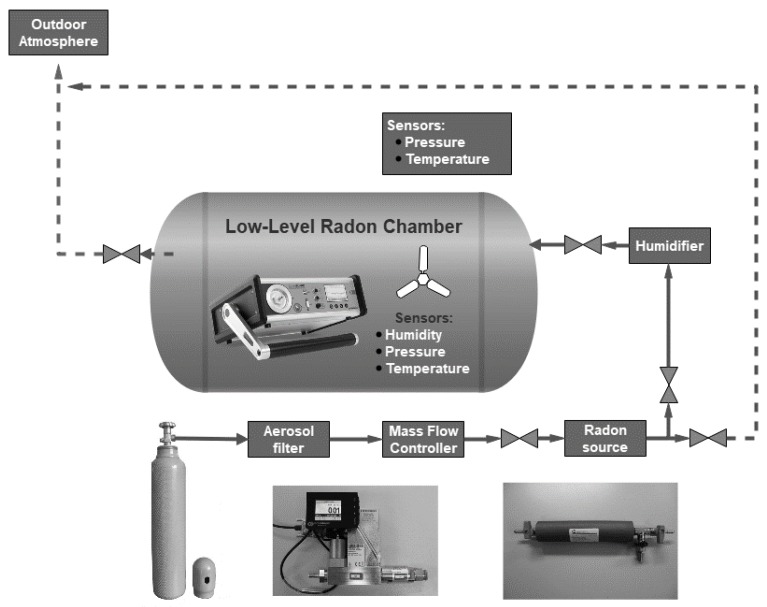
The simplified scheme of the equipment construction.

The newly developed equipment is a part of the Czech primary radon measurement device situated in SUJCHBO, v.v.i. Kamenna (Central Bohemia). Figure 1 represents a simplified scheme of the equipment construction. In particular, the equipment consists of an airtight low-level radon chamber (LLRCH), a humidifier, a flow-through source of Rn-222 type RF 5 (Prague, CZE), a mass flow controller of the Bronkhorst^®^ EL-Flow type (Bethlehem—PA, USA), an aerosol filter and an air pressure vessel as the source of radon-free air. 

In order to achieve a specific low-level radon concentration, it is necessary to ensure (1) a constant radon supply and (2) defined ventilation in the radon chamber. Because of the location of SUJCHBO, which is close to a former uranium mine, it is possible to measure an outdoor radon activity concentration in the range of tens or hundreds Bq·m^−3^. Therefore, it would not be possible to achieve a low-level radon activity concentration there without using a vessel with sufficiently radon-free air.

The air from the pressure vessel comes through the protective aerosol particle filter and the calibrated mass flow controller to the low-level radon source. The mixture of the air and radon comes through the humidifier to the radon chamber. The humidifier is installed into the construction to ensure that the measuring conditions are as real as possible.

The atmosphere homogeneity inside the radon chamber is ensured with the help of a continually regulated ventilator (the airflow speed can be set in the range 0.1–3.5 m·s^−1^). Sensors for the measurement of climatic conditions are placed inside the LLRCH. 

### 2.1. Reference Level of Radon

During the equipment design, a model of constant radon input and constant ventilation was applied:

(1) $$a\left(t\right)=a_{o}\cdot e^{-\left(\lambda +k\right)\cdot t}+\frac{R}{V\left(k+\lambda \right)}\left(1-e^{-\left(\lambda +k\right)\cdot t}\right)$$

where

a(t): radon activity concentration at time t (Bq·m^−3^);
$a_{o}$: radon activity concentration at time zero (Bq·m^−3^);
λ: radon decay constant (h^−1^);
k: air exchange intensity (h^−1^);
t: time (h);
R: radon input rate (Bq·h^−1^);
V: volume of radon chamber (m^3^).

For the steady state (t = ∞) at a constant air exchange intensity and constant radon input rate, the following applies:

(2) $$a_{V,\mathrm{Rn}}=R_{\mathrm{Rn}}/(Q_{\mathrm{settled}}\cdot \frac{M.p_{\;\mathrm{at}\;Q\;\mathrm{calibration}}}{R.T_{\;\mathrm{at}\;Q\;\mathrm{calibration}}}/\frac{M.p_{\;\mathrm{at}\;\mathrm{confrontation}}}{R.T_{\;\mathrm{at}\;\mathrm{confrontation}}}+\lambda_{\mathrm{Rn}}.V)$$

where

a_V,Rn_: radon activity concentration (Bq·m^−3^);
Q_settled_: flow rate (m^3^⋅h^−1^);
M: molar mass (kg·mol^−1^);
p _at Q calibration_ : air pressure 1013,25 (hPa);
R: molar gas constant (J·mol^−1^·K^−1^);
T _at Q calibration_ : temperature 273,16 (K);
p_at Rn confrontation_ : air pressure (Pa);
T_at Rn confrontation_ : temperature (K);
λ_Rn_ : radon decay constant (h^−1^);
V: volume of radon chamber (m^3^);
R_Rn_: radon emanation power (Bq·h^−1^).

### 2.2. Low-Level Radon Chamber

**Figure 2:**
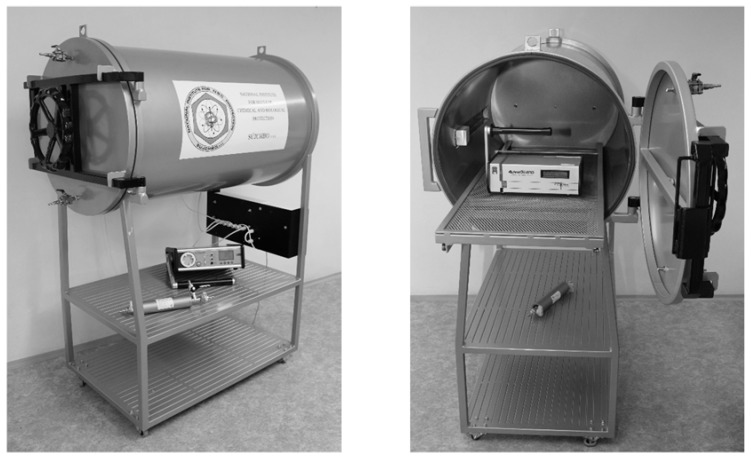
The low-level radon chamber in practice in the SUJCHBO, v.v.i. (National Institute for Nuclear, Biological and Chemical Protection) laboratory.

The low-level radon chamber (LLRCH) is made of steel, has a volume of 324 liters and has a cylindrical shape (Figure 2). The whole chamber is grounded, and the inner surface is painted with a special color to avoid or suppress the deposition of the radon decay products on the walls. 

The LLRCH is equipped with four sampling points for the connection of system components and to take samples of the inside air. These points are located in such a way that they allow sampling from different locations of the chamber.

The measuring device of climatic conditions enables the monitoring of temperature and air pressure by sensors placed inside and outside the radon chamber (the checking of possible under/overpressure with respect to natural air pressure in the laboratory) and the monitoring of relative humidity inside the radon chamber. A display reporting the on-line measured data is placed outside the chamber.

A movable drawer for the better manipulation of the used measuring devices is placed inside of LLRCH. 

During many experiments which required the adjustment of a required low-level activity concentration, the air-tightness of the LLRCH was verified. 

### 2.3. Low-Level Radon Source

In the 1950s, F. Kysela from the Institute for Research, Production and Utilization of Radioisotopes (ÚVVVR) in Prague created a primary set of 13 Ra-226 sources by filling RaBr_2_ into Thuringian glass tubes with a diameter of about 5 mm and wall thickness of 0.27 mm. Four of these emitters were compared to the standards of Prof. Otto Hoenigschmidt, deposited in Vienna, in 1957. The traceability was performed by Prof. Dr Berta Karlik from the Institut für Radiumforschung und Kernphysik. The remaining nine sources were compared to these four sources. The low-level radon emanation source used for the testing of LLRCH was created from one of the nine sources.

**Figure 3:**
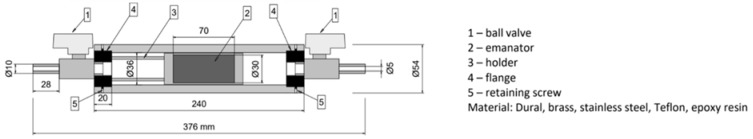
Scheme of the low-level radon source by the CMI (Czech Metrological Institute, Prague).

An emulsion of salts of fatty acids in silicone rubber was formed from the weighed standard solution. The emulsion was allowed to polymerize in a steel tray with the following dimensions: 70 × 30 mm. The activity of the standard as determined by the weight of the Ra-226 solution, the weight of the resulting emulsion and the losses (<0.1%). The whole process was controlled by weighing and gamma spectrometry on an HPGe detector. The 185 keV gamma-ray emission intensity was measured with the use of the standard solution, which confirmed excellent conformity with the tabulated value [6]. Figure 3 shows the scheme of the low-level radon source. The source was constructed as a stainless-steel cylindrical case, supplied on the ends with ball valves and two aerosol filters connected on the output aperture of the valves. The steel tray with Ra-226 was placed in the middle of this cylindrical case, and radon was released from this thin layer.

The emanation coefficient of the source was determined by measuring the activity of the Rn-222 daughter products (Pb-214/Bi-214) and the activity of Ra-226 and was almost equal to 1. The detection efficiency of the gamma photons was calculated by the MCNP code (Monte Carlo N-Particle Transport).

**Figure 4:**
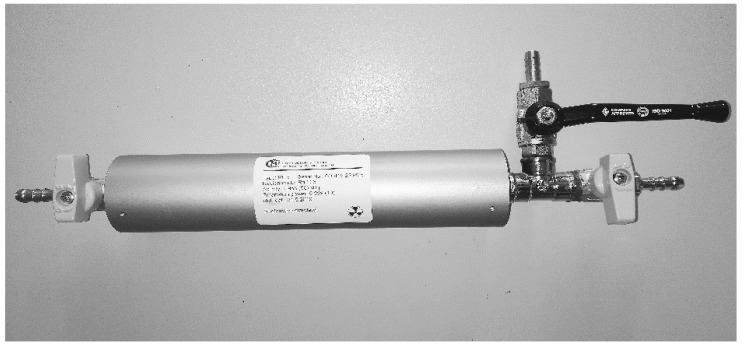
The flow-through low-level radon source developed by the CMI (Czech Metrological Institute, Prague).

The application of this source was the calibration of detectors for the activity measurements of Rn-222 in air. The source was operated in flow-through mode. Figure 4 shows the real photo of the radon source developed by the CMI (Czech Metrological Institute, Prague) with an original label.

The emanation power of radon from a radium source depends on the humidity of air flowing through the source vessel. The air coming from the vessel is ultra-dried, which was confirmed by the placing of a humidifier into the equipment (behind the radon source) and by measuring the relative humidity in the chamber with and without the humidifier being connected. If the humidifier was not connected, the relative humidity in the chamber was very close to zero. In case of the humidifier being connected, the relative humidity in the chamber was in the range of 40%–60% depending on the setting of the humidifier.

### 2.4. Mass Flow Controller

Bronkhorst^®^ model F-201CV (Bethlehem, PA, USA) mass flow controllers (MFCs) are suitable for the accurate measurement and control of flow ranges at operating pressures between a vacuum and 64 bar. The mass flow controller used in the equipment was calibrated by the Czech Metrological Institute. The MFC consists of a thermal mass flow sensor, a precise control valve and a microprocessor-based PC-board with signal and fieldbus conversion. The MFC is equipped with a digital high accuracy PC-board with a fast response. 

## 3. Results and Discussions

### 3.1. Experiments in LLRCH

**Figure 5:**
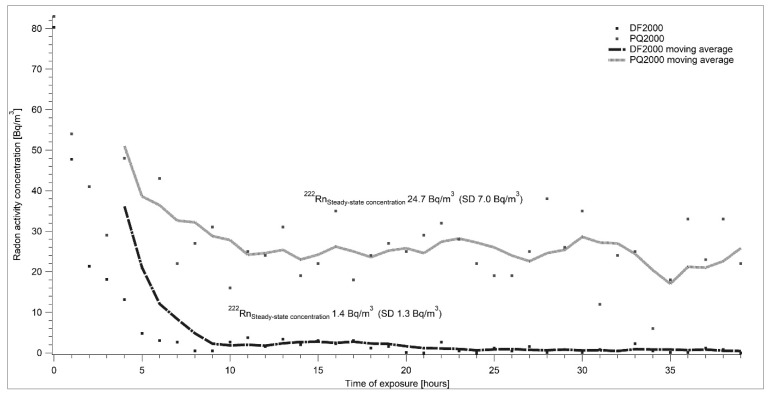
A stable radon-free atmosphere was set in approximately eight hours. Individual points show the real measured data and curves represent a moving average of measured results.

**Figure 6:**
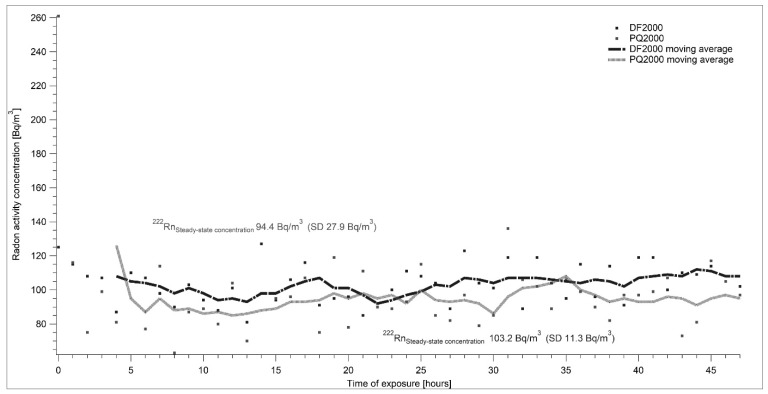
At the beginning of the experiment, the radon activity concentration in the laboratory was about 120 Bq·m^−3^. The flow rate was set to 6.20 l·min^−1^. The stable radon atmosphere at the level of 100 Bq·m^−3^ was set in approximately 6 h. Individual points show the real measured data and curves represent a moving average of measured results.

**Figure 7:**
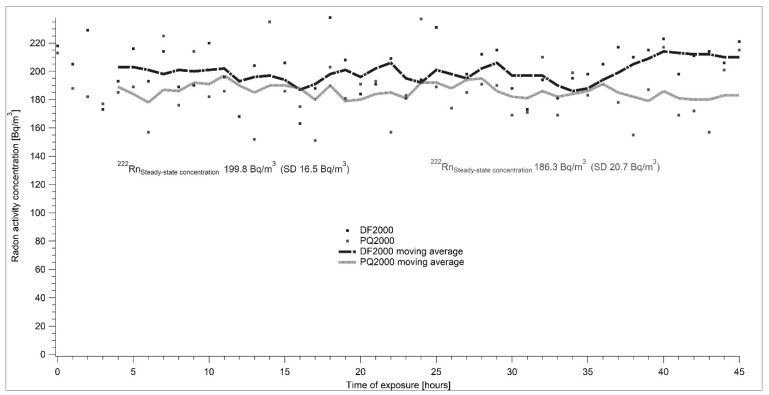
At the beginning of the experiment, at the required radon activity concentration of 200 Bq·m^−3^, the radon activity concentration in the laboratory was at a similar level. The flow rate was set to 3.08 l·min^−1^. The stable radon atmosphere was set almost immediately. Individual points show the real measured data and curves represent a moving average of measured results.

**Figure 8:**
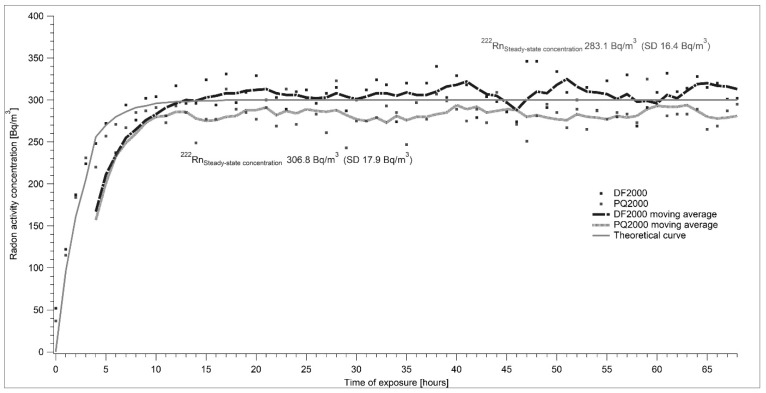
The flow rate in the experiment was set to 2.04 l·min^−1^. The stable radon activity concentration of 300 Bq·m^−3^ was set in approximately 10 h. Individual points show the real measured data and curves represent a moving average of measured results. The continuous line shows the theoretical radon activity trend obtained by applying Equation (2). The results of both measuring devices approximate the theoretical curve.

Two reference measuring devices—AlphaGuard DF 2000 and AlfaGuard PQ 2000—were used for the determination of radon activity concentration. Both devices are owned by SUJCHBO, v.v.i. and they were calibrated in BfS Berlin, Germany. During all experiments, the system was connected as shown in Figure 1. The flow-through source of Rn-222 type RF 5 with an emanation coefficient of 0.99 was used. Changes in the setting of the required level of radon activity concentrations were controlled by modifications of the flow rate through the radon source. Figure 5, Figure 6, Figure 7 and Figure 8 show the results of different settings of flow rates, the final required levels of radon activity concentrations in the LLRCH and the various times of radon activity concentration stabilizations. 

The graph shown in Figure 5 shows the results of the experiment in which the source of radon was not connected to the system. Only air free from radon was sent into the chamber. The goal of this experiment was to verify the air-tightness of the chamber and compare the background of measuring devices with data taken from calibration sheets. According to the Calibration sheets of both AlphaGuards, their backgrounds are 2.2 ± 1.2 Bq·m^−3^ for the DF 2000 model and 29.0 ± 7.0 Bq·m^−3^ for the PQ 2000 model. The measured average radon activity concentrations during the first experiment were 1.4 ± 1.3 Bq·m^−3^ with AhphaGuard DF 2000 and 24.7 ± 7.0 Bq·m^−3^ with AlphaGuard PQ 2000. The results of the experiment testify that zero radon activity concentration was maintained in the LLRCH for more than 30 h.

A set of experimental measurements was taken, demonstrating both the air-tightness of the low-level radon chamber and the capability of establishing and stably maintaining different radon concentrations. Radon reference atmospheres of 100, 200 and 300 Bq·m^−3^ (Figure 6, Figure 7 and Figure 8) were adjusted. The required radon activity concentrations were stabilized within hours, depending on the required radon activity concentration and related intensity of air exchange. The results were more than satisfying: for AlphaGuard DF 2000, the average radon activity concentrations were 103.2 ± 11.3 Bq·m^−3^, 199.8 ± 16.5 Bq·m^−3^ and 306.8 ± 17.9 Bq·m^−3^; for AlphaGuard PQ 2000, the average radon activity concentrations were 94.4 ± 27.9 Bq·m^−3^, 186.3 ± 20.7 Bq·m^−3^ and 283.1 ± 16.4 Bq·m^−3^. All results are presented after subtracting AlphaGuard´s background (according to the calibration sheets) and the calibration factor was used. Calibration factors for both AlphaGuards were determined in the framework of a unit traceability with the Czech Metrological Institute´s (CMI) primary standard of 226-Ra with the declared activity of 226-Ra.

The radon activity concentrations could be steadily maintained for several days depending on the amount of radon-free air available in the pressure vessel.

### 3.2. Radon Chamber Modelling

**Figure 9:**
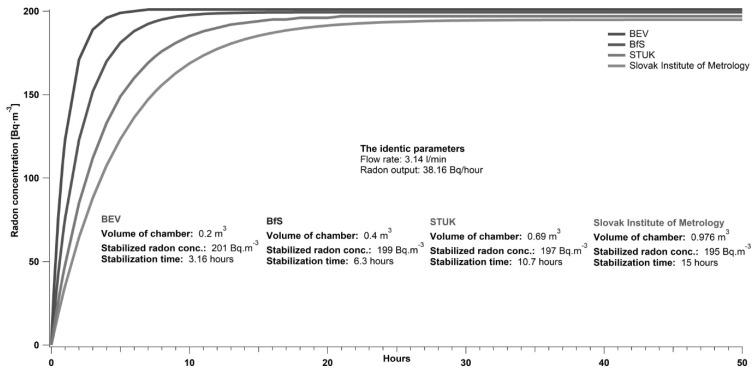
Theoretical radon activity concentration trend for a few selected existing radon chambers around the world.

The LLRCH with an inner volume of 324 l, placed in SUJCHBO, v.v.i. Kamenna, was developed and tested. However, the general system architecture can be applied to a different radon chamber size; for example, to the radon chambers mentioned in [7]. The radon activity concentration set in different chambers depends on the emanation power of a radon source and the flow rate of the air free from radon coming from a vessel to the radon source and then to the chamber, as in Equations (1) and (2). The time of radon activity concentration stabilization strongly depends on the volume of the chamber. Figure 9 shows the radon activity concentration trends obtained according to Equation (2) by modelling the radon chambers in use at the Federal Office of Metrology and Surveying (BEV, Austria), the Federal Office for Radiation Protection (BfS, Germany), the Radiation and Nuclear Safety Authority (STUK, Finland), and the Slovak Office of Standards, Metrology and Testing (Slovak Institute of Metrology, Slovak Republic). The volume data of the chambers were provided by the employees of individual institutions taking part in the Project 16ENV10 MetroRADON.

### 3.3. Uncertainties

The expanded uncertainty—the product of the standard measurement uncertainty and the expansion coefficient k = 2 (which corresponds to a coverage probability of about 95% for normal distribution) following the EA 04/02 [8]—was calculated as 2%.

## 4. Discussion and Conclusions

The low-level radon chamber of SUJCHBO, v.v.i. is a specific device developed for the calibration of measuring devices at a low-level radon activity concentration in the range of 100 Bq·m^−3^ to 300 Bq·m^−3^. Many tests have validated the air-tightness of the chamber and the possibility of adjusting a stable radon activity concentration to a required level for several days. A stable radon activity concentration is reached in several hours, depending on the required level of radon activity concentration and related intensity of air exchange. Generally, a higher required value of radon activity concentration means a lower intensity of air exchange and a much longer required time for stabilizing the radon atmosphere inside the chamber. 

The level of radon activity concentration in the LLRCH can be changed continuously during the experiment by an operator. The climatic parameters are continuously monitored by the sensors placed inside the chamber.

The low-level radon source of the CMI (Czech Metrological Institute, Prague) can be used for different radon chambers with a volume between 200 and 1000 liters.
